# Metabolic engineering of the mixed-acid fermentation pathway of *Escherichia coli* for anaerobic production of glutamate and itaconate

**DOI:** 10.1186/s13568-015-0147-y

**Published:** 2015-09-17

**Authors:** Kiira S. Vuoristo, Astrid E. Mars, Jose Vidal Sangra, Jan Springer, Gerrit Eggink, Johan P. M. Sanders, Ruud A. Weusthuis

**Affiliations:** Bioprocess Engineering, Wageningen University, P.O. Box 16, 6700 AA Wageningen, The Netherlands; Biobased Products, Wageningen University and Research Centre, P.O. Box 16, 6700 AA Wageningen, The Netherlands

**Keywords:** Itaconic acid, *Escherichia coli*, Metabolic engineering, Glutamic acid, Ethanol, Redox balance, Anaerobic fermentation

## Abstract

Itaconic acid, an unsaturated C5-dicarboxylic acid, is a biobased building block for the polymer industry. The purpose of this study was to establish proof of principle for an anaerobic fermentation process for the production of itaconic acid by modification of the mixed acid fermentation pathway of *E. coli*. *E. coli* BW25113 (DE3) and the phosphate acetyltransferase (*pta*) and lactate dehydrogenase (*ldhA*) deficient strain *E. coli* BW25113 (DE3) Δ*pta*-Δ*ldhA* were used to study anaerobic itaconate production in *E. coli*. Heterologous expression of the gene encoding *cis*-aconitate decarboxylase (*cadA)* from *A. terreus* in *E. coli* BW25113 (DE3) did not result in itaconate production under anaerobic conditions, but 0.08 mM of itaconate was formed when the genes encoding citrate synthase (*gltA*) and aconitase (*acnA*) from *Corynebacterium glutamicum* were also expressed. The same amount was produced when *cadA* was expressed in *E. coli* BW25113 (DE3) Δ*pta*-Δ*ldhA*. The titre increased 8 times to 0.66 mM (1.2 % Cmol) when *E. coli* BW25113 (DE3) Δ*pta*-Δ*ldhA* also expressed *gltA* and *acnA*. In addition, this strain produced 8.5 mM (13 % Cmol) of glutamate. The use of a nitrogen-limited growth medium reduced the accumulation of glutamate by nearly 50 % compared to the normal medium, and also resulted in a more than 3-fold increase of the itaconate titre to 2.9 mM. These results demonstrated that *E. coli* has potential to produce itaconate and glutamate under anaerobic conditions, closing the redox balance by co-production of succinate or ethanol with H_2_ and CO_2_.

## Introduction

Itaconic acid, an unsaturated C5 dicarboxylic acid produced by various microorganisms such as *Aspergillus terreus*, can be used as a precursor for many relevant compounds in chemical and pharmaceutical industries. It is especially of interest for the production of polymers, because of its potential as a substitute for acrylic and methacrylic acid (Okabe et al. 2009).

Current fermentation processes for the production of itaconic acid from sugar are executed aerobically using oxygen as the terminal electron acceptor (Kuenz et al. 2012). Aerobic processes result in higher operating and capital costs compared to anaerobic processes due to the lower yields and increased demands for oxygen and heat transfer (Cuellar et al. 2013; Zeikus 1980). Because of this, Zeikus (1980) stated that anaerobic fermentations form the basis for microbial production of chemicals and fuels. It is therefore interesting to design an anaerobic process for itaconic acid production.

The conversion of glucose to itaconate is an oxidation reaction that results in the net reduction of the NAD cofactor. Respiration is used to reoxidize NADH under aerobic conditions. Under anaerobic conditions alternative methods have to be employed for cofactor regeneration (Weusthuis et al. 2011).

*Escherichia coli* can produce itaconic acid under aerobic conditions when the *cis*-aconitate decarboxylase gene (*cadA*) from *Aspergillus terreus* is expressed (Li et al. 2011). We recently showed that itaconate production by *E. coli* is improved by enhancing the availability of precursors by overexpression of the first part of the citric acid cycle (citrate synthase (*gltA*) and aconitase (*acnA*) from *C. glutamicum* and reduction of the native metabolic routes to acetate and lactate by inactivating the genes encoding phosphate acetyltransferase (*pta*) and lactate dehydrogenase (*ldhA*) (Vuoristo et al. 2015).

**Fig. 1:**
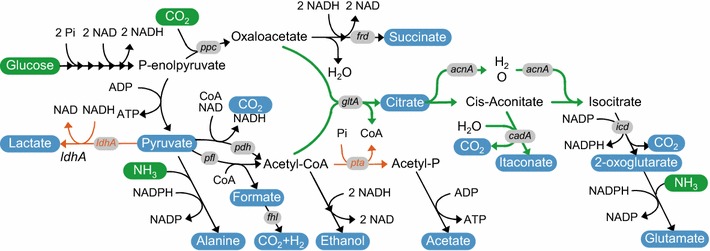
Anaerobic itaconate pathway in metabolically engineered *E. coli*. The *green bold arrows* indicate the introduced pathway consisting of genes encoding citrate synthase (*gltA*) and aconitase (*acnA*) from *C. glutamicum* and *cis*-aconitate decarboxylase (*cadA)* from *A. terreus*. The *red lines* indicate that phosphate acetyltransferase (*pta*) and lactate dehydrogenase (*ldhA*) were deleted

*Escherichia coli* is also one of the few industrial microorganisms that is able to grow under anaerobic conditions. It is therefore a suitable candidate to test whether anaerobic production of itaconic acid is possible. The mixed acid fermentation pathway of *E. coli* offers two options to regenerate NAD: the conversion of glucose and CO_2_ into succinate and the conversion of glucose into ethanol and formate (or hydrogen and CO_2_). The proposed heterofermentative pathway to itaconate and succinate or ethanol and formate/H_2_ is shown in Fig. 1.

In this paper, we cultivated *E. coli* strains under anaerobic conditions and analysed the effect of the introduction of *cis*-aconitate decarboxylase from *A. terreus* and citrate synthase and aconitase from *Corynebacterium glutamicum* on itaconate production, growth and formation of other fermentation products. Unexpectedly, strains started to produce significant amounts of glutamate when the itaconate pathway was introduced. Up to 2.9 mM of itaconate was produced when nitrogen-limited growth medium was introduced.

## Materials and methods

### Construction of pACYC expression vectors

All strains and plasmids used in this work are given in Table 1. The expression vector pKV-GA was derived from pKV-CGA by cloning the *acnA* and *gltA*-containing part of pKV-CGA in pACYC-Duet-1.

**Table 1:** *E. coli* strains and plasmids used in this study

Strains and plasmids	Characteristics	References
Strains		
BW25113 (DE3)	BW25113 DE3 T7 RNA polymerase	Vuoristo et al. (2015)
BW25113 (DE3) Δ*pta*Δ*ldhA*	BW25113 Δ*pta* Δ*ldhA* DE3 T7 RNA polymerase	Vuoristo et al. (2015)
BW25113 (DE3) Δ*pta*Δ*ldhA*Δ*icd*	BW25113 Δ*pta*Δ*ldhA*Δ*icd* DE3 T7 RNA polymerase	This study
Plasmids		
pKD46	AmpR plasmid with temperature-sensitive replication and arabinose induction of λ-red recombinase	The Coli Genetic Stock Center at Yale University (CGSC)
pKD13	KanR plasmid with R6K-γ replicon	CGSC
pCP20	AmpR and CmR plasmid with temperature-sensitive replicon and thermal induction of FLP synthesis	CGSC
pEV	pACYCDuet-1 expression vector using T7 promoter, with two multiple cloning sites, CmR	Novagen
pKV-C	pACYCDuet-1 derivative, synthetic *cadA*	Vuoristo et al. (2015)
pKV-CGA	pACYCDuet-1 derivative, synthetic *cadA*, *acnA*, and *gltA*	Vuoristo et al. (2015)
pKV-GA	pACYCDuet-1 derivative, synthetic *acnA*, and *gltA*	This study

### Cultivation conditions

#### Culture media

For plasmid construction, *E. coli* strains were cultured on Luria–Bertani (LB) agar plates or in LB liquid medium at either 30 °C or 37 °C. Recombinants harbouring temperature-sensitive plasmids were cultured at 30 °C for cultivation and at 42 °C to cure the selection markers. When needed, medium and plates were supplemented with ampicillin (50 μg/mL), kanamycin (50 μg/mL) or chloramphenicol (35 μg/mL). Induction of gene expression in liquid cultures was started by the addition of 1 mM of IPTG when the optical density at 600 nm (OD_600_) of the culture was approximately 0.4.

The other cultivations were done either in M9 minimal medium (MM) or in nitrogen-limited minimal medium (NL-MM) with chloramphenicol (35 μg/mL). MM contained per 1 L: 200 mL 5 × M9 minimal salts (BD Difco) supplemented with 50 mM of glucose, 2 mM of MgSO4, 0.1 mM of CaCl_2_, 15 mg of thiamine, and 0.30 mg of selenite. Medium was buffered with 0.1 M 3-(*N*-morpholino) propanesulfonic acid (MOPS) and the pH was adjusted to 6.9 with NaOH. The nitrogen limited medium (NL-MM) contained 0.5 g/L NH_4_Cl, which is 50 % less than in standard M9. In some cultivations, MM and NL-MM were supplemented with US* trace elements (Panke et al. 1999), yielding MM* and NL-MM*, respectively, to reduce the lag phase.

### Cultivation in bioreactors

*Escherichia coli* BW25113 (DE3) and *E. coli* BW25113 (DE3) Δ*pta*-Δ*ldhA* containing either pEV, pKV-C or pKV-CGA were cultivated at 30 °C in 0.5 L Mini Bioreactors, connected to myControl controller units (Applikon, The Netherlands) with a working volume of 400 mL. The pH was maintained at 6.9 by the automated addition of 2 M NaOH. Cultures were stirred at 400 rpm and sparged with nitrogen at 16 mL/min for 17 h, after which the stirring speed was increased to 800 rpm and the sparging rate was increased to at 35 mL/min. Bioreactor cultures that were grown in MM* or NL-MM* were stirred at 500 rpm and sparged with air at 150 mL/min for 4 h, followed by nitrogen sparging at 35 mL/min. Bioreactors were inoculated with 5 % (v/v) of a pre-culture that was grown at 30 °C in a 250 mL Erlenmeyer flasks with 50 mL of MM at 250 rpm for 24 h. Samples of 2 mL were regularly taken to determine the OD_600_ of the cultures and the concentrations of substrate and products. The product distribution (% Cmol) was calculated by dividing the amounts of products formed by the amount of substrate consumed after 72 h of cultivation.

### Deletion of isocitrate dehydrogenase (*icd*) gene

The gene encoding isocitrate dehydrogenase (*icd*) was inactivated in *E. coli* BW25113 (DE3) Δ*pta*-Δ*ldhA* by using the Lambda red-mediated gene replacement method described by Datsenko and Wanner (2000). Shortly, *E. coli* BW25113 (DE3) Δ*pta*-Δ*ldhA* was transformed with pKD46 and cultured in the presence of L-arabinose to induce λ-red recombinase expression, which is an inducer for recombination. The target gene *icd* was replaced by a kanamycin-resistance gene flanked by flippase recognition target (*FRT*) sites. For this, a deletion cassette containing a kanamycin-resistance gene with *FRT* sites was amplified from pKD13 by using Phusion High Fidelity DNA Polymerase (Thermo Scientific) and primers that contain 50 bp targeting flanks to the *icd* region in the genome (Table 2), and transformed into *E. coli* BW25113 (DE3) Δ*pta*-Δ*ldhA* (pKD46). Transformants were screened for their proper genotype by selecting for kanamycin resistance and colony PCR (GoTaq Green polymerase, Promega) using primers that flank the target gene. The phenotype was verified in liquid cultures and by sequencing. The kanamycin-resistance gene was subsequently eliminated by using the temperature-sensitive helper plasmid pCP20 encoding the flippase (*FLP*), followed by curing of the temperature sensitive plasmids by culturing strains at 42 °C for 16 h.

**Table 2:** List of primers used in this study

Name and description	Sequence
For *icd* deletion	
icd flank F	CCCGTTAATAAATTTAACAAACTACGGCATTACATGTTTTCGATGATCGCTGTAGGCTGGAGCTGCTTCG
icd flank R	AACGTGGTGGCAGACGAGCAAACCAGTAGCGCTCGAAGGAGAGGTGAATGATTCCGGGGATCCGTCGACC
For PCR verifications	
icd check F	ACGTGGTGGCAGACGAGCAAAC
icd check R	TTAATAAATTTAACAAACTACGG
pACYC MCS1 F	GGATCTCGACGCTCTCCCT
pACYC MCS1 R	GATTATGCGGCCGTGTACAA
Sequencing	
icd200seqF	GGATCACACGCGTGGGCTG
icd200seqR	GGTGTAAGGAGTGGTAATTCA

The 50 bp targeting flanks are underlined

The presence of the 1.5 kB *cadA* region in *E. coli* BW25113 (DE3) Δ*pta*-Δ*ldhA*Δ*icd* that was transformed with either pKV-C or pKV-CGA were analysed by PCR using GoTaq Green DNA polymerase (Promega) and primers pACYC MCS1 F and pACYC MCS2 R (Table 2).

### Analytical methods

The cell density was determined by measuring the OD_600_ by using a spectrophotometer (Dr. Lange XION 500). For CDW determination, 100 ml of fermentation medium was centrifuged (7745×*g*, 10 min), and the pellet was washed with 0.7 % (w/v) NaCl. The pellet was resuspended in deionized water and dried at 100 °C until constant weight. The relation between OD and CDW was found to be CDW [g/L] = 0.25 × OD600. The molar ratio of CH_1.83_O_0.5_N_0.22_P_0.017_, which corresponds to a molecular weight of 24.63 g per mol C was used to determine the amount of C in biomass.

The concentrations of glucose, ethanol and organic acids were determined by using HPLC by using a Dionex Ultimate 3000 (Thermo Fisher) equipped with an RI detector (Shodex, RI-101) and a UV detector (Dionex, 3400 RS at 210 nm). The samples were separated on a Micro Guard Cation H pre-column (30 × 4.6 mm, Biorad) and an Aminex HPX-87H column (300 × 7.8 mm, Biorad) at 35 °C, using 0.6 mL/min of 5 mM H_2_SO_4_ as eluent.

The concentrations of glutamate and alanine were determined by using UPLC Dionex RSLC system with an UltiMate 3000 Rapid Separation pump as described by Meussen et al. (2014). Glutamate concentrations were also determined by using l-Glutamic Acid Assay Kit (K-GLUT07/12, Megazyme).

The concentrations of CO_2_ and H_2_ in the off-gas of the bioreactors were determined by using BlueSens Off-Gas Sensors (Gas Sensor, GmbH).

## Results

### Itaconate production under anaerobic conditions

**Fig. 2:**
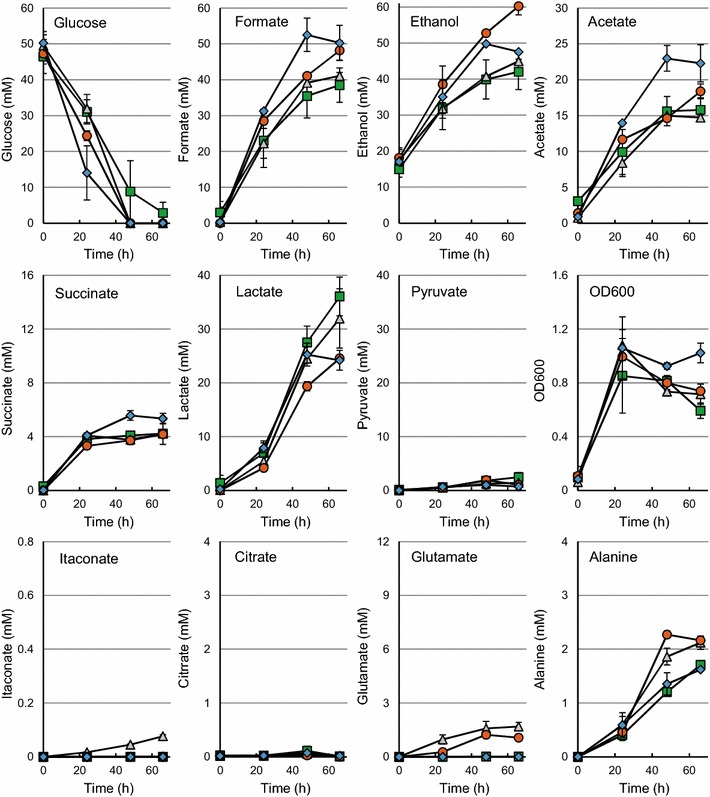
Anaerobic cultivation of *E. coli* BW25113 (DE3) containing pEV (*diamonds*), pKV-C (*squares*) pKV-CGA (*triangles*) or pKV-GA (*circles*) in pH-controlled bioreactors on MM at 30 °C. The average values of duplicate cultures are given. Standard deviations are based on replicas of two parallel cultivations

*Escherichia coli* BW25113 (DE3) and *E. coli* BW25113 (DE3) Δ*pta*-Δ*ldhA* containing pEV. pKV-C or pKV-CGA were grown on M9 minimal medium (MM) at pH 6.9 in pH-controlled bioreactors under anaerobic conditions with glucose as carbon source. The main fermentation products of *E. coli* BW25113 (DE3) (pEV) were lactate, ethanol, formate and acetate (Fig. 2), which accounted for 74 % of the carbon that was added to the culture (Table 3). As a lot of formate (16 % Cmol) was formed in *E. coli* BW25113 (DE3) (pEV), only low amounts of CO_2_ (3 mmol/L, <1 % Cmol) and H_2_ (12 mmol/L) were produced. The formation of acetate was redox balanced with the co-production of ethanol and succinate.

**Table 3:** Product distribution in  % Cmol in culture supernatants of *E. coli* BW25113 (DE3) and *E. coli* BW25113(DE3) Δ*pta*-Δ*ldhA* containing pEV, pKV-C, pKV-CGA or pKV-GA after 66 h in pH-controlled bioreactors on MM at 30 °C

Strains and plasmids	Formate	Ethanol	Acetate	Succinate	Lactate	Pyruvate	Itaconate	Citrate	Glutamate	Alanine	Biomass	CO_2_	C-recovery %
*E. coli* BW25113 (DE3)													
pEV	15.8	20.4	12.8	6.8	26.7	1.4	0.0	0.0	0.0	1.7	3.0	0.9	89.5
pKV-C	14.3	26.2	11.4	5.3	33.5	2.3	0.0	0.5	0.0	1.5	2.2	1.4	98.6
pKV-CGA	13.5	18.7	9.2	5.9	37.9	1.9	0.1	0.0	2.5	2.3	2.0	1.1	95.0
pKV-GA	17.9	27.4	13.0	7.0	25.9	1.1	0.0	0.0	1.7	2.2	2.4	0.9	99.4
*E. coli* BW25113 (DE3) Δpta-ΔldhA													
pEV	0.0	23.0	10.4	18.0	0.1	20.2	0.0	2.7	0.2	3.7	1.8	14.4^a^	94.4
pKV-C	0.0	29.7	5.2	17.6	0.1	36.0	0.1	1.5	0.3	3.5	3.2	13.2	110.3
pKV-CGA	0.0	29.8	3.3	12.1	0.8	17.9	1.2	4.0	13.0	3.6	2.8	16.4	104.9
pKV-GA	0.1	24.9	3.7	11.0	0.3	13.8	0.0	5.1	19.5	4.2	3.2	15.4	101.2

The average values of duplicate cultures are given

^a^Based on theoretical CO_2_ production

**Fig. 3:**
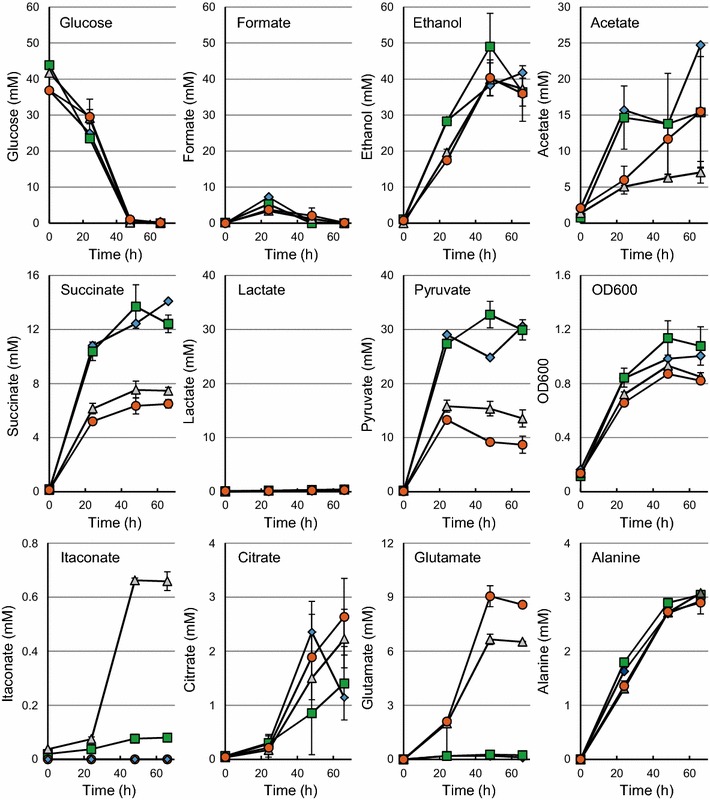
Anaerobic cultivation of *E. coli* BW25113 (DE3) Δ*pta*-Δ*ldhA* containing pEV (*diamonds*), pKV-C (*squares*) pKV-CGA (*triangles*) or pKV-GA (*circles*) in pH-controlled bioreactors on MM at 30 °C. The average values of duplicate cultures are given. Standard deviations are based on replicas of two parallel cultivations

*Escherichia coli* BW25113 (DE3) Δ*pta*-Δ*ldhA* (pEV), in which *pta*, encoding phosphate acetyltransferase, and *ldhA,* encoding lactate dehydrogenase were eliminated, still produced acetate in comparable amounts as *E. coli* BW25113 (DE3) (pEV), but lactate was no longer formed (Fig. 3; Table 3). *E. coli* BW25113 (DE3) Δ*pta*-Δ*ldhA* (pEV) did not produce formate. Instead, the production of CO_2_ (30 mmol/L, 14 % Cmol) and H_2_ (>100 mmol) were both more than 10 times higher than observed with *E. coli* BW25113 (DE3) (pEV) and the amount of succinate was doubled. Also high amounts of pyruvate and some citrate accumulated in the culture.

pKV-C and pKV-CGA both express codon-optimized *cadA*, which encodes the *cis*-aconitate decarboxylase from *Aspergillus terreus* that was previously shown to enable the production of itaconate in *E. coli* [5] (Vuoristo et al. 2015). pKV-CGA also expresses citrate synthase (*gltA*) and aconitase (*acnA*) from *Corynebacterium glutamicum*. These genes enhanced the production of itaconate in *E. coli* BW25113 (DE3) Δ*pta*-Δ*ldhA* under aerobic conditions (Vuoristo et al. 2015).

Expression of *cadA* did not result in itaconate production in *E. coli* BW25113 (DE3) (pKV-C) (Fig. 2), but 0.08 mM of itaconate was produced by *E. coli* BW25113 (DE3) Δ*pta*-Δ*ldhA* (pKV-C) (Fig. 3). A similar amount of itaconate was formed by *E. coli* BW25113 (DE3) (pKV-CGA) (Fig. 2). *E. coli* BW25113 (DE3) Δ*pta*-Δ*ldhA* (pKV-CGA) produced eight times more itaconate (Fig. 3), showing that both the expression of *gltA* and *acnA* and the elimination of *pta* and *ldhA* stimulate the production of itaconate in *E. coli* under anaerobic conditions.

The fermentation products that were formed by the strains carrying either pKV-C or pKV-CGA were similar to those formed by the strains carrying pEV. However, the carbon recovery for strain *E. coli* BW25113 (DE3) Δ*pta*-Δ*ldhA* (pKV-CGA) was initially much lower (84 %) than for the other strains, indicating that unidentified products were formed. Amino acid analysis of the culture supernatants showed that this strain accumulated significant amounts of glutamate and alanine. Alanine was also produced by all other strains but glutamate was only produced in large amounts by strain *E. coli* BW25113 (DE3) Δ*pta*-Δ*ldhA* (pKV-CGA). This indicates that expression of *gltA* and *acnA* increased the flux through the citric acid cycle, resulting in more itaconate and the accumulation of glutamate (Fig. 3). Carbon balances for all strains were satisfactory (90–110 %) when the production of glutamate and alanine was taken into account (Table 3).

### Glutamate production under anaerobic conditions

To further investigate the production of glutamate under anaerobic conditions, pKV-GA (*gltA* and *acnA* were expressed without *cadA*) in *E. coli* BW25113 (DE3) and *E. coli* BW25113 (DE3) Δ*pta*-Δ*ldhA*. The latter strain produced eight times more glutamate than the former one, resulting in the excretion of 8.6 mM of glutamate. This accounts for 19.5 % of the carbon that was added to the culture.

### Elimination of isocitrate dehydrogenase (*icd*)

*Escherichia coli* BW25113 (DE3) Δ*pta*-Δ*ldhA* strains produced significant amounts of glutamate under anaerobic conditions when *gltA* and *acnA* from *C. glutamicum* were expressed. Glutamate is produced in *E. coli* via the citric acid cycle suggesting that citrate and *cis*-aconitate are more efficiently channelled through the citric acid cycle than towards itaconate formation. To prevent isocitrate conversion to 2-oxoglutarate and further to glutamate, isocitrate dehydrogenase (*icd)* was deleted from *E. coli* BW25113 (DE3) Δ*pta*-Δ*ldhA.* This resulted in the glutamate auxotrophic strain *E. coli* BW25113 (DE3) Δ*pta*-Δ*ldhA*-Δ*icd*. Growth of this strain was hampered under anaerobic conditions (OD_600_ <0.5 after 66 h). When pKV-CGA and pKV-C were transformed into this strain, PCR analyses with cells of colonies that were grown on LB plates with chloramphenicol (35 μg/mL) for 16 h after transformation yielded fragments of 1.5 kB, indicating that *cadA* was present. However, all transformants lost a part of *cadA* during 16 h cultivation on liquid LB medium as PCR analysis with these cultures as template yielded fragments of only 0.3 kB (data not shown). The loss of a part of *cadA* was also already occurring in several colonies on the LB plates, as some PCR reactions yielded fragments of both 1.5 and 0.3 kB. Different ways to improve the stability of *cadA* like altered media and growth conditions were tried without success. The results indicate that *E. coli* BW25113 (DE3) Δ*pta*-Δ*ldhA*-Δ*icd* is not able to maintain a complete *cadA*.

### Itaconate production under nitrogen limited conditions

**Fig. 4:**
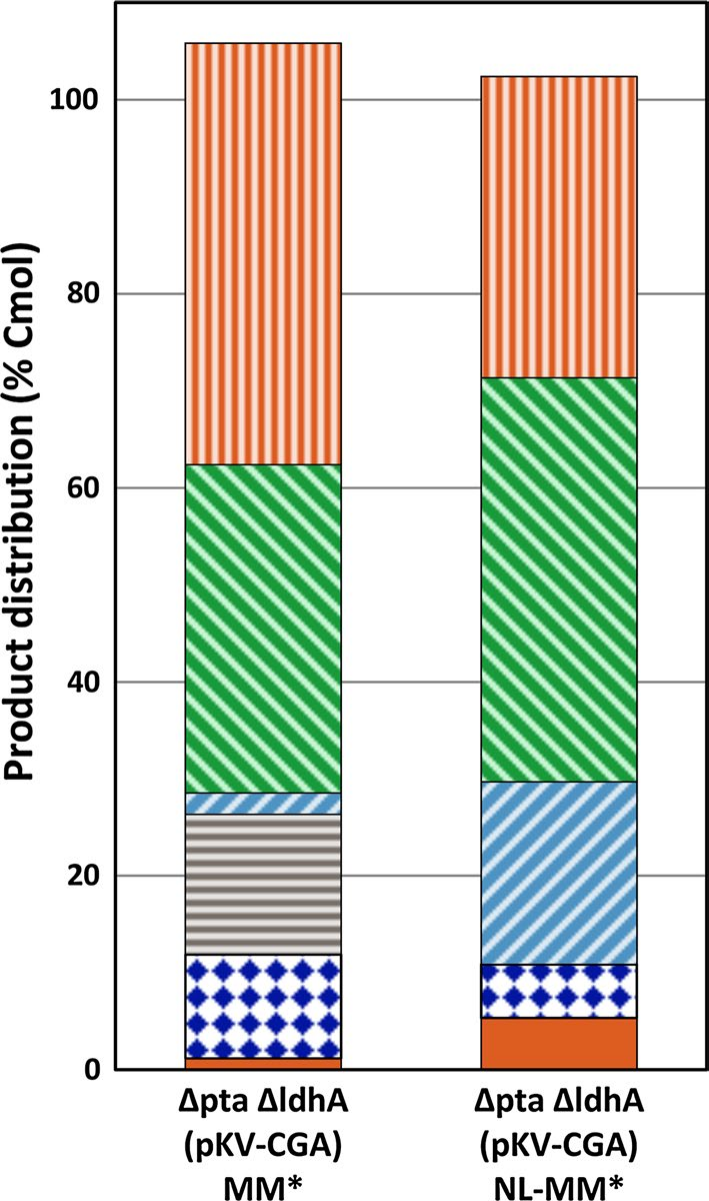
Product distribution of bioreactor cultures of *E. coli* BW25113 (DE3) Δ*pta*-Δ*ldhA* (pKV-CGA) cultivated in MM* and NL-MM* after 72 h. Itaconate (*solid*), glutamate (*diamonds*), citric acid (*horizontal stripes*), pyruvate (*upward diagonal stripes*), ethanol (*downward diagonal stripes*), and other products (*vertical stripes*). The average values of duplicate cultures are given

Itaconate production in *E. coli* BW25113 (DE3) Δ*pta*-Δ*ldhA*-Δ*icd* turned out to be impossible due to the partial loss of *cadA*. Another option to prevent glutamate formation is to limit the availability of ammonium, as it is required for glutamate synthesis. This strategy was tested in bioreactors by using medium in which the amount of nitrogen was reduced to 50 %. Besides, US* trace elements were added to the medium, and a short aerobic growth phase was added to fermentation scheme to reduce the length of the lag phase. The use of nitrogen-limited medium resulted in a 50 % reduction of the production of glutamate and a 3-fold increase of the itaconate yield (Fig. 4). The itaconate titre of the latter culture was 2.9 mM.

## Discussion

We have previously constructed an *E. coli* strain that is able to produce itaconate under aerobic conditions by overexpressing *cis*-aconitate decarboxylase (*cadA*) from *A. terreus*. The flux to itaconate was enhanced by overexpressing the genes encoding citrate synthase (*gltA*) and aconitase (*acnA*) from *C. glutamicum* and eliminating the genes encoding phosphotransacetylase (*pta*) and lactate dehydrogenase (*ldhA*) (Vuoristo et al. 2015). Under anaerobic conditions, *E. coli* uses a mixed acid fermentation in which various products like acetate, succinate, ethanol, formate, lactate, hydrogen and carbon dioxide are synthesized (Clark 1989). The fluxes to these products and to biomass in *E. coli* is combined in such a way that a redox balance is maintained. Itaconate production, as well as acetate and pyruvate production—results in cofactor reduction, which can be balanced by the co-production of succinate and/or ethanol. In this study we investigated whether it is possible to realize anaerobic production of itaconate in *E. coli*.

The *E. coli* strains that were previously developed for aerobic production of itaconate were cultivated under anaerobic conditions. Expression of *cadA* in *E. coli* BW25113 (DE3) was not sufficient to initiate itaconate production. Citrate synthase from *E. coli* is controlling the flux through the citric acid cycle because it is allosterically inhibited by the high NADH concentrations that occur under anaerobic conditions, and this may have prevented the production of itaconate. The citrate synthase from *C. glutamicum* (GltA) is not affected by such a negative feedback (Eikmanns et al. 1994). Indeed, co-expression of *acnA* and *gltA* from *C. glutamicum* in *E. coli* BW25113 (DE3) had a positive effect on itaconate production as it resulted in itaconate production, but the titres were low.

As the itaconate titre and yield were low, it is important to prevent unnecessary by-product formation. Knocking out *ldhA* completely suppressed lactate production, but the elimination of *pta* did not result in a significant reduction of acetate production. A similar observation was made under aerobic conditions (Vuoristo et al. 2015) and confirms the existence of alternative pathways for acetate production.

Expression of *cadA* in *E. coli* BW25113 (DE3) Δ*pta*Δ*ldhA* was already sufficient to evoke itaconate production, but the strain also accumulated pyruvate and citrate, indicating that the pathway to itaconate was restrained. Pyruvate accumulation is likely caused by a redox imbalance. To maintain redox balance, *E. coli* BW25113 (DE3) Δ*pta*Δ*ldhA* has to produce itaconate together with ethanol and/or succinate. When the flux to itaconate is too low compared to the fluxes to ethanol and succinate, the strains will become NADH-limited, which resulted in pyruvate accumulation. Additional expression of *gltA* and *acnA* from *C. glutamicum* strongly stimulated itaconate production and reduced the amount of pyruvate that was formed, resulting in an 8 times increased titre of itaconate.

We earlier showed that heterologous expression of *cadA* leads to inclusion body formation (Vuoristo et al. 2015). Strategies to increase the solubility of CadA in *E. coli* such as laboratory-directed protein evolution (Yuan et al. 2005) or codon harmonization (Angov et al. 2008) are likely to increase the flux from aconitate. Another option is that intracellular concentration of itaconate and the lack of transport capacity might become rate limiting, which was also proposed by Okamoto et al. (2014). DauA was characterized as the main succinate transporter in *E. coli*, but it was shown to transport also other dicarboxylic acids at pH 7 (Karinou et al. 2013), suggesting that overexpression of *dauA* may boost itaconate export. Several putative itaconate transporters have recently been characterized in *Aspergillus* species (Li et al. 2011; van der Straat et al. 2014), but their functionality *in E. coli* has not been tested.

Unexpectedly, glutamate was produced in *E. coli* BW25113 (DE3) Δ*pta*Δ*ldhA* cultures in which *gltA* and *acnA* of *C. glutamicum* were expressed. The UPLC method used to determine glutamate also revealed that significant amounts of alanine were produced (1.5–3 % C-mol) in all *E. coli* strains. Apparently, alanine is a standard fermentation product of this *E. coli* strain. Literature search did not reveal other studies in which alanine was found as a standard fermentation product of *E. coli*.

Both itaconate and glutamate synthesis compete for the same intermediates. To increase the flux to itaconate it is therefore necessary to repress glutamate production. Glutamate auxotrophs of *E. coli* have been realized by knocking out the genes encoding either citrate synthase (*gltA*) (Mainguet et al. 2013), aconitase (*acnA*) (Gruer et al. 1997) or isocitrate dehydrogenase (*icd*) (Lakshmi and Helling 1976). *GltA* and *acnA* are involved in itaconate production and are therefore unsuitable candidates. Both *icd* and *acnA* knockouts are known to be instable under aerobic conditions as they lead to inactivation of *gltA*, possibly because of a toxic effect of intracellularly accumulating citrate (Gruer et al. 1997). Still, Gruer et al. (1997) showed that *acnA* knockouts were stable under anaerobic conditions, which suggests that less citrate accumulates under anaerobic conditions, possibly because of the regulation of the activity of GltA by NADH. Introduction of the NADH-insensitive citrate synthase of *C. glutamicum* in *E. coli* BW25113 (DE3) ∆*pta*∆*ldhA*∆*icd* may therefore not be feasible. Indeed, attempts to express the genes of pKV-CGA in the strain were unsuccessful. Even expression of pKV-C, which only contains *cadA*, resulted in the loss of a part of *cadA* during growth, which suggests that the instability of *icd* knockouts may also be caused by the accumulation of high intracellular itaconate concentrations.

In a recent study (Okamoto et al. 2014), CadA was successfully expressed in ∆*icd* strain when cultured on LB medium under aerobic conditions. Overexpression of aconitase *(acnB)* together with *cadA* in the ∆*icd* strain led to enhanced itaconate production (4.34 g/L). However, a complex growth medium like LB, which seemed to stabilize expression of *cadA* in ∆*icd* background, is not preferred for bulk chemical production due to its high price. In addition, the ∆*icd* strain accumulated a substantial amount of acetate without a deletion in metabolic pathways involved in acetate metabolism, such as *pta,* and the authors recommended to inactivate the acetate forming pathways. In another study, Icd activity of *C. glutamicum* was lowered by exchanging the ATG start codon to GTG or TTG, which together with a heterologous CadA expression resulted in 60 mM of itaconate (Otten et al. 2015).

As glutamate production depends on the availability of nitrogen in the medium, an alternative strategy to diminish glutamate production was tested by culturing cells in nitrogen-limited medium. This enhanced the production of itaconate to up to 5.4 % Cmol with *E. coli* BW25113 (DE3) Δ*pta*Δ*ldhA* pKV-CGA. Enhancing the flux from aconitate to itaconate would probably further reduce the amount of glutamate production.

*Escherichia coli* has several interesting features for anaerobic production of itaconate: It is one of the few industrial microorganisms that is able to grow under anaerobic conditions. Acetyl-CoA—a precursor of itaconate—is a central metabolite in dissimilation processes in *E. coli*, which is not the case in eukaryotes like *Saccharomyces cerevisiae*—although several groups are trying to change this (Kozak et al. 2014; Lian et al. 2014). *E. coli* converts pyruvate into acetyl-CoA and formate by pyruvate-formate lyase under anaerobic conditions. Formate can subsequently be split into valuable H_2_ and CO_2_. Other industrial strains that are able to grow under anaerobic conditions, like *S. cerevisiae* and lactic acid bacteria, use NAD-dependent pyruvate dehydrogenase to synthesize acetyl-CoA, which generates extra NADH and thus requires additional cofactor regeneration at the cost of substrate.

The microbial production of organic acids is studied by many groups [see e.g. Wendisch et al. (2006) and Yu et al. (2011)]. Addition of base (lime) is necessary when organic acids are produced at neutral pH. During downstream processing the organic acid salt has to be converted into the organic acid, which is usually done by adding sulphuric acid. This results in the production of vast quantities of salts (gypsum). An alternative approach is to produce the organic acid at low pH. *E. coli* is unable to grow at low pH values and organic acid production with *E. coli* can therefore only be done at neutral pH values.

This study shows that it is possible to synthesize itaconate anaerobically by using the mixed acid pathway of *E. coli*, in which the synthesis of ethanol/H_2_ and succinate regenerate NAD. Ethanol/H_2_ seems to be the best set of co-products for industrial application as these products can be simply separated based on their boiling temperatures, and easily marketed as bulk chemicals. Itaconate and succinate are more difficult to separate because they are both dicarboxylic acids with a C4 backbone. Moreover they may copolymerize, which will have an impact on polymer properties. Deleting fumarate reductase (*frd*) is one of the obvious solutions to prevent succinate formation (Zhou et al. 2006).

This is the first time that anaerobic production of itaconate from glucose was reported for *E. coli*. The observed yields and productivities are still modest. Eliminating the pathways to major by-products like glutamate, succinate, and acetate, and enhancing the pathway between pyruvate and itaconate is therefore crucial to obtain a cost-competitive anaerobic production process for itaconic acid.
